# Supplementary material for: Antirheumatic treatment is associated with reduced serum Syndecan-1 in Rheumatoid Arthritis
Source: PLoS One. 2021 Jul 9;16(7):e0253247. doi: 10.1371/journal.pone.0253247 (PMC8270157; doi:10.1371/journal.pone.0253247)
Supplement: Attachment — Submitted filename: Rebuttal letter.docx [file pone.0253247.s002.docx]

# Rebuttal letter

**Reviewer 1**: The present is an interesting study, aiming to evaluate impact of different kind of medications on mediators of atheroslerosis in RA.

*Comment 1*: Reasons for use of TNFi should at least summarized

*Response 1:* This is addressed in the section “Material and methods –Patients” at page 6, line 7 where the following is described: In PSARA study, the treatment regimen was chosen using clinical judgment by rheumatologists not involved in the study, and in accordance with the Norwegian treatment guidelines. All patients starting with MTX were MTX naïve, and all patients starting with TNFi had used MTX earlier without sufficient effect. This is also described in Hjeltnes et al (1) and this reference is already included in the manuscript at page 7 line 6.

*Comment 2*: The authors speak about multiple regression analysis. Do they means logistic regression? cox multivariate analysis (even if the follow up was the same).

*Response 2*: Thank you for pointing out this editing error. We have changed the text from “multiple regression analysis” to “multiple **linear** regression analysis” throughout the whole manuscript.

*Comment 3*: The authors reported B (which in this case probably is OR) and also negative values for CI>should they perform loghartimis transformation to report in a more readable way?

*Response 3*: We report betas (estimated regression coefficients) from linear regression, not from logistic regression. Thus, the betas can be interpreted as the effect on the dependent variable of one unit increase in the independent variable. The scale of the betas in linear regression are from minus infinity to infinity, with zero as the value of no effect.

*Comment 4*: Should authors perform a multivariate analysis for change in baseline syndecan?

*Response* *4*: In the manuscript under section Results, “**Predictors of change in syndecan-1, TIMP-1 and MMP-9”,** page 10**,** we presented both simple and multiple linear regression analyses with baseline syndecan-1 as the dependent variables, and several factors as independent variables.

**Reviewer 2**: Thank you for your effort. However, this study is quite observational and includes very small number of samples, and seems to lack scientific quality to consider publication in PLOS ONE.

*Comment 1*: Compared to the wide variation of serum syndecan-1 levels at baseline, its change seems quite marginal (although statistically significant). It is difficult for readers to understand whether this minor change leads to cardioprotective effects.

*Response 1*: Several studies support the notion of a role of syndecans in cardiovascular disease. However, the evidence is still limited, and exact cut-offs for clinically significant changes in syndecan levels have not been identified yet. In our previous prospective population-based study, we found an association between syndecan-shedding and cardiovascular endpoints (2). This is addresses in the manuscript page 13, line 26.
Also, we found an association between syndecan 1-shedding and endothelial function (3). In both of these studies we observed a variation in baseline levels of syndecan-1, in line with what we observed in this present study. Accordingly, it is important to interpret results from this study with caution, keeping in mind the relative low

sample size.

However, this explorative study is important as no other similar studies exist, and as it was able to detect statistically significant differences and associations in spite of the limited sample size. It can help to plan further studies (e.g. by facilitating sample size calculations for future studies).

*Comment 2*. There are other factors deeply involved in atherosclerosis of RA such as TNF-α. Were there any correlation with serum syndecan-1 levels and these factors?

*Response 2*: We agree with the point made by the referee. We did perform simple linear regression analyses with baseline syndecan-1, TIMP -1 and MMP-9, respectively, as the dependent variables, and several independent variables (please refer to section Results, “Predictors of baseline levels of syndecan-1, TIMP-1 and MMP-9”, page 10 in the manuscript). Further we performed simple and multiple linear regression analyses examining relationships between changes in syndecan-1, TIMP-1 or MMP-9 as dependent variables and age, gender and change in the following independent variables: CRP, ESR, IL-6, TNF, DAS28, number of swollen joints, rheumatoid factor, anti-citrullinated antibodies, MHAQ, RHI, ED, E-selectin, ICAM-1, VCAM-1, Apo B-1, LDL-C, HDL-C, total cholesterol, serum glucose and HbA_1_c. There was no correlation between syndecan-1 and TNF and other factors besides CRP in simple linear regression analysis and LDL-c in multiple linear regression analysis (see table 3 in the manuscript).

*Comment 3:* The quality of the figure is poor and difficult to read.

*Response 3:* Thank you for pointing this out. We have provided a figure with better quality.

*Comment 4:* Tables should be inserted between the main manuscript but not at last. Please read the instruction carefully.

*Response 4*: Thank you for your comment. The manuscript is now in accordance with PlosOne instructions.

**References**

1.	Hjeltnes G, Hollan I, Førre O, Wiik A, Lyberg T, Mikkelsen K, et al. Serum levels of lipoprotein(a) and E-selectin are reduced in rheumatoid arthritis patients treated with methotrexate or methotrexate in combination with TNF-α-inhibitor. Clin Exp Rheumatol. 2013;31(3):415-21.

2.	Solbu MD, Kolset SO, Jenssen TG, Wilsgaard T, Løchen ML, Mathiesen EB, et al. Gender differences in the association of syndecan-4 with myocardial infarction: The population-based Tromsø Study. Atherosclerosis. 2018;278:166-73.

3.	Lee S, Kolset SO, Birkeland KI, Drevon CA, Reine TM. Acute exercise increases syndecan-1 and -4 serum concentrations. Glycoconj J. 2019;36(2):113-25.
